# Massive Pulmonary Embolism at the Onset of Acute Promyelocytic Leukemia

**DOI:** 10.4084/MJHID.2016.027

**Published:** 2016-07-01

**Authors:** Federica Sorà, Patrizia Chiusolo, Luca Laurenti, Francesco Autore, Sabrina Giammarco, Simona Sica

**Affiliations:** Istituto di Ematologia, Università Cattolica del Sacro Cuore di Roma, Roma

## Abstract

Life-threatening bleeding is a major and early complication of acute promyelocytic leukemia (APL), but in the last years there is a growing evidence of thromboses in APL. We report the first case of a young woman with dyspnea as the first symptom of APL due to massive pulmonary embolism (PE) successfully treated with thrombolysis for PE and heparin. APL has been processed with a combination of all-trans retinoic acid (ATRA) and arsenic trioxide (ATO) obtaining complete remission.

## Case report

Bleeding is the usual manifestation of acute promyelocytic leukemia (APL) related coagulopathy and is associated with significant morbidity and mortality.^1^–^3^ Thrombocytopenia, hyperfibrinolysis and disseminated intravascular coagulation (DIC) are the leading causes of bleeding at diagnosis and shortly after the introduction of treatment.^4^ On the contrary thrombotic complications were considered rare before the introduction of ATRA. As a result, while the APL-related bleeding diathesis has been investigated extensively and the pathogenesis has been elucidated to a greater extent, the importance of thrombosis in APL, as a cause of morbidity and mortality, has been undervalued and scarcely studied. So, the exact pathogenetic mechanism of thrombosis in APL is still a matter of controversy, even if the incidence of thrombotic complications in APL seems to be rising. This could simply be due to higher levels of vigilance and availability of advanced diagnostic modalities, or, due to the increasing use of ATRA as suggested by Rashidi et al in a very exhaustive review of this topic.^5^–^7^ There are several potential explanations for thromboembolic complications in APL, including: (i) A mere coincidence (e.g. genetic predisposition, prolonged bed rest or immobility). (ii) Thrombogenicity of APL cells. (iii) The use of all-trans retinoic acid (ATRA). (iv) The combination of ATRA and antifibrinolytic agents, and the differentiation syndrome caused by ATRA (i.e. ATRA syndrome).^4^ We here present the case of a young patient admitted to our hospital with a massive pulmonary embolism as the first sign of acute promyelocytic leukemia requiring thrombolysis and heparin. Finally, this case offers a snapshot of the kinetic of APL related coagulopathy on therapeutic heparin during ATRA and ATO and suggests the role of ATO and ATRA in the treatment of APL patients with major thrombosis at diagnosis.

## Methods and Result

A 24-year-old Caucasian female was admitted to the emergency department of a peripheral hospital because of a few days history of progressive shortness of breath. She also experienced an increasing asthenia, uncommon for a young girl. Physical examination showed pallor in the absence of bleeding, lymphadenopathy or hepatosplenomegaly. She was afebrile and had only mild fluid retention. Vital signs including blood pressure and heart rate were normal. Respiratory rate was 20 breaths per minute and O_2_ saturation by pulse oximetry was 96% with a FiO_2_ 0.35 support by face mask. Her blood test revealed normocytic normochromic anemia (Hb 7.7 g/dl) severe leucopenia and neutropenia (WBC 800/mm^3^, ANC 480) with a platelet count of 109,000/mm^3^. Peripheral smear did not show abnormal cells. Biochemistry values and coagulation profile were normal. Chest X-Ray revealed ground-glass opacity in right upper lobe.

The patient was referred 48 hours later to our Division of Hematology. Sh complained of shortness of breath requiring oxygen supplementation and she had two episodes of hemoptysis. Her past medical history was unremarkable except for adrenogenital syndrome requiring long-standing estroprogestinic support and coeliac disease. She denied medication allergies, use of alcohol, cigarettes or illicit substances. Neutropenia and anemia with a platelet count of 110,000/mm^3^ were confirmed and coagulation profile was normal except for d-dimer 7283 ng/ml (normal values <300ng/ml). International Society on Thrombosis and Hemostasis (ISTH) DIC score was 4.^8^

Bone marrow aspiration was carried out showing a massive infiltration of pathologic promyelocytes and faggot cells consistent with APL. These cells were markedly positive to myeloperoxidase. Flow cytometry demonstrated an extensive positivity for CD117, myeloperoxidase (MPO), CD45, CD13, CD33, and partial for CD34 and absence of HLA-DR, CD2 expression. CD15 was not tested. Fluorescence in situ hybridization (FISH) revealed that 94% of nuclei were positive for t(15;17). Cytogenetics showed a normal karyotype 46XX (20 cells). PML/RAR alpha chimeric protein, bcr3 type, was detected by reverse transcriptase-polymerase chain reaction (RT-PCR) from leukemic blasts. FLT3 was positive.

Computerized tomography of the chest revealed a massive acute pulmonary embolism with large filling defects of both main arteries also involving lobar, segmental and subsegmental arteries. There were also peripheral wedge-shaped areas of hyperattenuation representing infarcts in many different lobes and a minimal pericardial effusion was detected. The patient was started immediately on intravenous sodium heparin and ATRA (45mg/m^2^ per day in two divided doses orally )with prophylactic methylprednisolone (0.5mg/kg) to prevent the differentiation syndrome. 10 However, few hours later her general condition deteriorated with hypotension, tachypnea (respiratory rate 32 breaths per minute) and hypoxia with an oxygen saturation of 80% requiring a progressive increase of FiO_2_ up to 1. Arterial blood gasses showed pH 7.477, pCO_2_ of 23.3 mm Hg, and pO_2_ of 59 mm Hg. Alteplase was given at the dose of 100 mg over two hours according to the recent guidelines from AACP.^9^ Sodium heparin was titrated in order to obtain 1.5 times the baseline control value of the activated partial thromboplastin time (aPTT). Bedside echocardiogram demonstrated mild right ventricular (RV) hypertrophy with normal global kinesis. Pulmonary artery pressure (PAPS) was estimated to be 31 mmHg. Lower limbs venous color Doppler ultrasonography did not reveal signs of deep or superficial thrombosis. Inherited and acquired thrombophilia screening tests were negative.

After thrombolysis, her pulmonary function dramatically improved.

**Figure 1:**
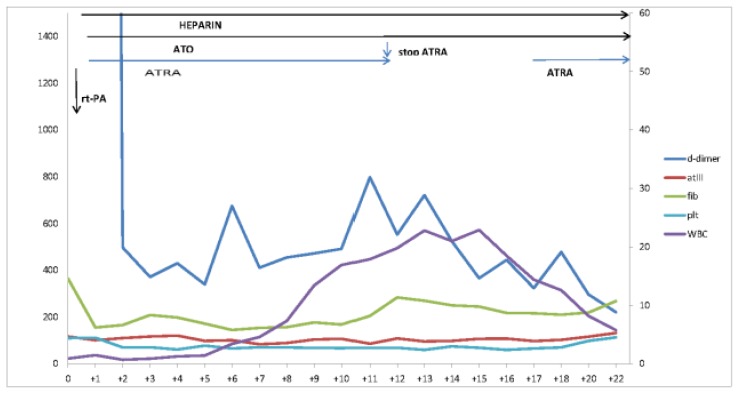
White Blood Cell Count, fibrinogen, D-Dimer, platelet value during first time treatment.

At the second day of ATRA, considering the diagnosis of low-risk APL^10^ and the requirement of long-term anticoagulation, arsenic trioxide (ATO) at the standard dose of 0.15mg/Kg/die was added.^11^ At the third day, breath rate was normal and oxygen supplementation was discontinued. On day +11 of ATO+ATRA she developed mild chest pain, asthenia and a slight increase in troponin levels and a T wave abnormality on the ECG; chest CT scan was unchanged raising the suspicion of a differentiation syndrome. ATRA was discontinued for five days and dexamethasone 10mg bid was given until the complete resolution of the symptoms. Leukocytosis, defined as a white-blood-cell count (WBC) of more than 10×109 per liter, developed at day+ 17 and was successfully managed with hydroxyurea.^11^ The kinetic of coagulative parameters, including platelets and WBC count during induction with ATO and ATRA, is depicted in figure 1.

After 50 days of ATRA and ATO peripheral blood cell count and bone marrow biopsy documented the complete morphological and molecular remission of APL and the patient was discharged from the hospital on low molecular weight heparin. CT showed a slight reduction of EP defects and the areas of hyperattenuation representing infarcts. Echocardiogram described normal kinesis and valvular function with a slight increased PAPS (35 mmHg). Consolidation therapy with ATRA and ATO was delivered.^11^ The patient is currently alive and well after the end of ATO consolidation and in molecular CR at 20 months from diagnosis and off anticoagulation.

## Discussion

Thrombotic events (TE) appear to be more common in acute promyelocytic leukemia (APL) than in other acute leukemias, with reported incidence ranging from 2 to 10–15%. Data from large cooperative groups have been published after initial reports of increased thrombotic events in APL when a combination of ATRA and chemotherapy was applied.^12^ The incidence of thrombosis in APL patients was 8.8% in AIDA protocol from the GIMEMA group^13^–^14^ and 5.1% in PETHEMA LPA96 and LPA99 Protocol,^15^ occurring mainly at diagnosis or during induction.^1^ In the GIMEMA study risk factors for the development of thrombosis included higher leukocyte count, the prevalence of bcr3 transcript type and a malignant cell phenotype including CD2 and CD15. ATRA-induced differentiation syndrome was associated with a severe increase of thrombotic complications in the PETHEMA group experience. These data were partially confirmed in a recent paper from Mitrovic et al^16^ in a retrospective study of 63 APL patients. The authors reported an incidence of thrombotic complications of 20.6%, including asymptomatic thrombosis, and again more frequently observed during induction.

In our case massive PE developed at diagnosis. No APL-specific risk factors for thromboses or inherited or acquired thrombophilia were found in this particular case except for bcr3 isoform and estroprogestinic exposure. Despite the concurrent diagnosis of APL, the patient required both heparin and thrombolysis for PE. Two other attempts to treat major thromboses with thrombolysis were reported in APL, one patient was treated for Budd-Chiari syndrome^17^ and relapsed APL and a more recent one APL patient received local thrombolysis for arterial thrombosis at the onset of APL.^18^ One additional patient underwent to thrombolysis for PE when in remission of APL.^19^ The combination of ATRA and ATO, adopted in considering the low-risk APL category of our patient and the need for prolonged and sustained anticoagulation, demonstrated to be an efficient regimen, also significantly reducing the risk of prolonged thrombocytopenia due to chemotherapy. Two additional cases with severe thrombotic events at diagnosis successfully treated with anticoagulants with or without thrombolysis when in ATO and ATRA have been published recently^18^,^20^ and reported in Table 1.

Although this case is exceptional, being to the best of our knowledge the only reported case of thrombolysis for massive PE attempted in APL at diagnosis, it raises several points of discussion Thrombosis should be kept in mind for observation and treatment of APL patients as suggested by a growing body of evidence.^1^ An accurate evaluation of risks should be pursued and anticoagulation including thrombolysis might be necessary for APL at least in catastrophic thrombosis. Although the management with ATRA and ATO in induction, is largely anecdotal in APL patients anticoagulated, with thrombosis and concurrent risk of bleeding, avoiding chemotherapy might allow adequate anticoagulation by reducing the length and the severity of thrombocytopenia. In literature now the results of some trials^21^,^22^ support the not inferiority of ATRA/ATO-based regimen in “all-risk” acute promyelocytic leukemia regarding efficacy and safety; these data and our experience suggest this combination also in high-risk APL patients submitted to anticoagulation.

## Figures and Tables

**Table 1:** Cases with APL and concomitant severe thrombotic events at diagnosis successfully treated with ATO and ATRA + Anticoagulant with/without Thrombolysis

	sex	Age years	Time from symptoms to diagnosis	Site of thrombosis	Anticoagulant treatment	APL treatment	Leukemia response
Trottie-Tellier-et al	female	52	2 weeks	DVT right leght then Axillary, subclavia and ulnary arteries	Thrombolysis and argatroban	ATRA+ATO	CR
Vaid at al	male	48	2 weeks	DVT and PE	LowMolecular Weight Heparin	ATRA+ATO	CR
Sorà et al	female	24	1 weeks	DVT and PE	Thrombolysis and sodium heparin	ATRA+ATO	CR
